# Genome Sequence of Sulfide-Dependent Denitrification Bacterium *Thermomonas* sp. Strain XSG, Isolated from Marine Sediment

**DOI:** 10.1128/MRA.00057-21

**Published:** 2021-04-15

**Authors:** Xiao-Tong Wu, Yu-Qin He, Guo-Xiang Li, Hang Xiao, Xiao-Rong Dai, Meng-Rong Yang, Peng Bao

**Affiliations:** a Key Lab of Urban Environment and Health, Institute of Urban Environment, Chinese Academy of Sciences, Xiamen, People’s Republic of China; b Ningbo Urban Environment Observation and Research Station, Chinese Academy of Sciences, Ningbo, People’s Republic of China; c College of Life Sciences, Fujian Agriculture and Forestry University, Fuzhou, Fujian, People’s Republic of China; d University of Chinese Academy of Sciences, Beijing, People’s Republic of China; e Center for Applied Geosciences (ZAG), Eberhard Karls University Tuebingen, Tuebingen, Germany; Georgia Institute of Technology

## Abstract

We report the draft genome sequence of *Thermomonas* sp. strain XSG, isolated from a marine sediment. The genome is 3,047,478 bp long with a GC content of 68.5%. Strain XSG was found to be closely related to strain NBRC 101115 of Thermomonas koreensis.

## Abstract (precis)

We report the draft genome sequence of *Thermomonas* sp. strain XSG, isolated from a marine sediment. The genome is 3,047,478 bp long with a GC content of 68.5%.

## ANNOUNCEMENT

The genus *Thermomonas*, belonging to the family *Xanthomonadaceae* branch within the *Gammaproteobacteria*, was first proposed by Busse et al. in 2002 (1). Currently, the genus *Thermomonas* includes 7 species with validly published names (https://lpsn.dsmz.de/genus/thermomonas) (2). Members of the genus *Thermomonas* have been isolated from a wide diversity of habitats, such as kaolin slurry (1), a hot spring (3), a denitrification reactor (4), a ginseng field (5), the soil of a coal mine (6), and an industrial wastewater treatment plant (7). The genus *Thermomonas* is considered to be part of an abundant group of denitrification bacteria in ecosystems. The DNA G+C content of strains of species of the genus *Thermomonas* ranges from 64.7 to 68.7% (1, 3–7).

In the present study, we report the whole-genome sequence of *Thermomonas* sp. strain XSG, isolated from a marine sediment (29°31′21″N, 121°36′59″E) at Xiangshan Harbor, Ningbo, Zhejiang, People’s Republic of China.

The sample was collected at a depth of 14 m in January 2018. We attempted to isolate *Thermomonas* sp. XSG using the enrichment method (8). Genomic DNA was extracted using the Mag-MK bacterial genomic DNA extraction kit (Songon Biotech, People’s Republic of China), and extracted DNA was used for both Nanopore and Illumina sequencing. The long reads for *Thermomonas* sp. XSG were generated with PromethION sequencing (Oxford Nanopore Technologies). The genomic DNA was sheared to generate fragments for long-insert library preparation. The BluePippin system (Sage Science, Beverly, MA) was used to size select DNA fragments. The sequencing library was prepared using a ligation sequencing kit (SQK-LSK109) and was run in an R9.4.1 flow cell. Base calling and demultiplexing were performed using Guppy v3.2.6. For the Illumina sequencing, the library constructed using the NEBNext Ultra DNA library prep kit for Illumina was sequenced using the Illumina NovaSeq platform (150-bp paired-end reads). A total of 106,844 and 72,265,892 reads were obtained for the Nanopore and Illumina sequencing, respectively. Fastp v0.20.0 (9) was used to filter the raw Illumina reads and remove adapters. The Nanopore reads were adapter trimmed and quality controlled using the MinKNOW software package, which contains Albacore v1.1.2 and Guppy v3.2.6, and were corrected using Canu v1.8.0 (10). The genome sequence was obtained by *de novo* hybrid assembly using SPAdes v3.14.1 (11). The final assembly was polished using Pilon v1.23 (12). Default parameters were used for all software unless otherwise noted.

The assembly resulted in one circular chromosome with a total length of 3,047,478 bp and a G+C content of 68.5%. The average sequence depths were 275.6× (Nanopore) and 3,238.18× (Illumina). Annotation was performed using the Prokaryotic Genome Annotation Pipeline (PGAP) (13). From a total of 2,861 predicted sequences, 2,786 and 56 were protein and RNA coding sequences, respectively. To assess similarity, 16S rRNA gene sequences were searched using BLAST against the NCBI sequence database. Strain XSG exhibited 16S rRNA gene sequence similarity of 99.86% to the strain NBRC 101115 of Thermomonas koreensis. Our data suggest that the strain XSG is a member of the genus *Thermomonas*. The strain was temporarily classified as *T. koreensis*.

### Data availability.

This whole-genome project has been deposited in GenBank under the accession number CP061497.1. The raw sequencing data have been deposited in the SRA under BioProject accession number PRJNA661730.

## References

[1] Busse HJ, Kampfer P, Moore ERB, Nuutinen J, Tsitko IV, Denner EBM, Vauterin L, Valens M, Rossello-Mora R, Salkinoja-Salonen MS. 2002. *Thermomonas haemolytica* gen. nov., sp. nov., a gamma-proteobacterium from kaolin slurry. Int J Syst Evol Microbiol 52:473–483. doi:10.1099/00207713-52-2-473. 11931159

[2] Parte AC, Sarda Carbasse J, Meier-Kolthoff JP, Reimer LC, Goker M. 2020. List of Prokaryotic names with Standing in Nomenclature (LPSN) moves to the DSMZ. Int J Syst Evol Microbiol 70:5607–5612. doi:10.1099/ijsem.0.004332. 32701423 PMC7723251

[3] Alves MP, Rainey FA, Nobre MF, da Costa MS. 2003. *Thermomonas hydrothermalis* sp. nov., a new slightly thermophilic gamma-proteobacterium isolated from a hot spring in central Portugal. Syst Appl Microbiol 26:70–75. doi:10.1078/072320203322337335. 12747412

[4] Mergaert J, Cnockaert MC, Swings J. 2003. *Thermomonas fusca* sp. nov. and *Thermomonas brevis* sp. nov., two mesophilic species isolated from a denitrification reactor with poly(epsilon-caprolactone) plastic granules as fixed bed, and emended description of the genus *Thermomonas*. Int J Syst Evol Microbiol 53:1961–1966. doi:10.1099/ijs.0.02684-0. 14657130

[5] Kim MK, Im WT, In JG, Kim SH, Yang DC. 2006. *Thermomonas koreensis* sp. nov., a mesophilic bacterium isolated from a ginseng field. Int J Syst Evol Microbiol 56:1615–1619. doi:10.1099/ijs.0.64049-0. 16825638

[6] Wang L, Zheng S, Wang D, Wang L, Wang G. 2014. *Thermomonas carbonis* sp. nov., isolated from the soil of a coal mine. Int J Syst Evol Microbiol 64:3631–3635. doi:10.1099/ijs.0.063800-0. 25070215

[7] Ju JH, Kim JS, Lee DH, Jeon JH, Heo SY, Seo JW, Kim CH, Park DS, Oh BR. 2019. *Thermomonas aquatica* sp. nov., isolated from an industrial wastewater treatment plant. Int J Syst Evol Microbiol 69:3399–3404. doi:10.1099/ijsem.0.003630. 31380735

[8] Li G-X, Li H, Xiao K-Q, Bao P. 2020. Thiosulfate reduction coupled with anaerobic ammonium oxidation by Ralstonia sp. GX3-BWBA. ACS Earth Space Chem 4:2426–2434. doi:10.1021/acsearthspacechem.0c00267.

[9] Chen S, Zhou Y, Chen Y, Gu J. 2018. Fastp: an ultra-fast all-in-one FASTQ preprocessor. Bioinformatics 34:i884–i890. doi:10.1093/bioinformatics/bty560. 30423086 PMC6129281

[10] Koren S, Walenz BP, Berlin K, Miller JR, Bergman NH, Phillippy AM. 2017. Canu: scalable and accurate long-read assembly via adaptive k-mer weighting and repeat separation. Genome Res 27:722–736. doi:10.1101/gr.215087.116. 28298431 PMC5411767

[11] Bankevich A, Nurk S, Antipov D, Gurevich AA, Dvorkin M, Kulikov AS, Lesin VM, Nikolenko SI, Pham S, Prjibelski AD, Pyshkin AV, Sirotkin AV, Vyahhi N, Tesler G, Alekseyev MA, Pevzner PA. 2012. SPAdes: a new genome assembly algorithm and its applications to single-cell sequencing. J Comput Biol 19:455–477. doi:10.1089/cmb.2012.0021. 22506599 PMC3342519

[12] Walker BJ, Abeel T, Shea T, Priest M, Abouelliel A, Sakthikumar S, Cuomo CA, Zeng Q, Wortman J, Young SK, Earl AM. 2014. Pilon: an integrated tool for comprehensive microbial variant detection and genome assembly improvement. PLoS One 9:e112963. doi:10.1371/journal.pone.0112963. 25409509 PMC4237348

[13] Tatusova T, DiCuccio M, Badretdin A, Chetvernin V, Nawrocki EP, Zaslavsky L, Lomsadze A, Pruitt KD, Borodovsky M, Ostell J. 2016. NCBI Prokaryotic Genome Annotation Pipeline. Nucleic Acids Res 44:6614–6624. doi:10.1093/nar/gkw569. 27342282 PMC5001611

